# Reply to S Eggener, M Gonzalgo and O Yossepowitch: ‘High-intensity-focused ultrasound in the treatment of primary prostate cancer: the first UK series’

**DOI:** 10.1038/sj.bjc.6605456

**Published:** 2009-12-08

**Authors:** H U Ahmed, M Emberton

**Affiliations:** 1 Division of Surgery and Interventional Sciences, University College, London, UK; 2 NIHR University College Hospital/UCL Comprehensive Biomedical Research Centre, London, UK

**Sir,**

We are grateful to Eggener *et al* (2009) for their interest in our article on using high-intensity-focused ultrasound (HIFU) to treat localised prostate cancer (Ahmed *et al*, 2009). We agree that existing treatments are unlikely to achieve the ideal trifecta of low incontinence, low erectile dysfunction and good cancer control. This is despite the trend towards increased specification and costs (intensity-modulated radiotherapy; robotic/laparoscopic radical prostatectomy). It is for this reason that we began to explore HIFU as a possible alternative.

We are grateful to Eggener *et al* for commending us on our efforts. We have attempted to report all outcomes, both functional and cancer control, in as robust a manner as the dataset allowed. Indeed, to this end, we chose a number of definitions of biochemical failure and analysed functional outcomes at a number of thresholds so as to ensure that readers were not misled with respect to patient-reported outcome measures and that the denominator patient group was looked at. These efforts were all in order to allow the reader to derive the true value and place of HIFU in the treatment of prostate cancer. We do reiterate that this was not a prospective study but represents an important cohort in that they were the first to be treated within the UK. As a result, our series has a number of interventions (the conduct of the HIFU evolved); it contains a spectrum of risk from as low as T1c to as high as T3a, as well as numerous learning curves. It therefore represents what could be achieved in a pragmatic real-practice setting. We did our best to explain this so that the reader was aware of the limitations of the study.

However, one should not underestimate the value of a treatment that can be delivered in a day-case setting, be repeated and deliver very low rates of incontinence for a disease in which 48 men need to be treated in order to save the life of one man over 10 years (Schroder *et al*, 2009). We agree that the impotence rates were not as encouraging as we would have liked. Indeed, our focus at present is on using the attributes of HIFU to treat in a focal manner with phase II prospective ethically approved trials close to completion (Ahmed *et al*, 2007;  , 2009). This seems to us and many others to be the most plausible way in which we can achieve the ‘perfect result’ for the majority of men who are currently subject to much harm.
